# Noradrenaline Regulation of Tyrosine Hydroxylase Expression in Arcuate Nucleus Neurons in Young and Adult Rats

**DOI:** 10.3390/ijms27167308

**Published:** 2026-08-16

**Authors:** Tatiana S. Pronina, Dmitry V. Troshev, Michael V. Ugrumov

**Affiliations:** Laboratory of Neural and Neuroendocrine Regulations, Koltzov Institute of Developmental Biology of the Russian Academy of Sciences, Moscow 119334, Russia; tatiana.pronina@mail.ru (T.S.P.); dmitry.vad.troshev@gmail.com (D.V.T.)

**Keywords:** arcuate nucleus, tyrosine hydroxylase, noradrenaline, adrenoreceptors, young rats, adult rats, Western blotting, real-timePCR

## Abstract

Neurons of the arcuate nucleus (AN) produce dopamine, which inhibits prolactin secretion. Tyrosine hydroxylase (TH), the key enzyme of dopamine synthesis, is expressed in AN in dopaminergic neurons and in neurons expressing only TH or both enzymes but lacking the dopamine transporter. These neurons are distributed differently between the ventrolateral and dorsomedial regions of the AN (further—ventrolateral or dorsomedial AN). We hypothesized that noradrenaline released by noradrenergic afferents inhibits TH synthesis in AN neurons postnatally. To test this hypothesis, we assessed: (i) adrenoreceptors gene expression in the ventrolateral and dorsomedial AN of intact rats at postnatal days (P) 5 and 60 and (ii) TH levels in sections of each AN region from rats at P5 and P60 after 6 h incubation in the absence or presence of noradrenaline, as well as noradrenaline with adrenoreceptor antagonists. It was shown that (i) AN neurons express genes for all types of adrenoreceptors in each region of AN on P5 and P60; (ii) neurons in both regions of AN synthesize TH, but to a greater extent at P60 than at P5; and (iii) noradrenaline inhibits TH synthesis, but only in the ventrolateral AN at P60—this action is mediated via α1-adrenoreceptors.

## 1. Introduction

The functioning of the endocrine hypothalamus, including the arcuate nucleus (AN) and its neural and humoral regulations, have attracted the attention of researchers for nearly half a century [1,2,3]. It has been shown that AN is involved in the neuroendocrine regulation of most adenohypophysial functions and, through the adenohypophysis, in the regulation of peripheral organs, including endocrine glands [4]. This is due to the fact that the AN contains neurons that synthesize adenohypophysotropic neurohormones, so-called releasing hormones and inhibiting hormones [1,2,5].

One of the most functionally important adenohypophysiotropic neurohormone of the AN is dopamine, which inhibits prolactin secretion via type 2 dopamine receptors [3,6]. In the 1980s, neurons containing tyrosine hydroxylase (TH), the first enzyme of dopamine synthesis, were discovered in the AN using monoimmunolabeling on histological sections, the only available immunohistochemical approach at that time. These neurons were mistakenly considered to be dopaminergic [7,8]. After the development of the double-immunolabeling method, it was shown that the AN, in addition to dopaminergic neurons, contains non-dopaminergic neurons that express TH or TH and aromatic L-amino acid decarboxylase (AADC), but lack the dopamine transporter [9].

Neurons expressing TH were found to be distributed in the AN with a specific zonality. Neurons expressing both dopamine-synthesizing enzymes and only TH are localized in both the dorsomedial and ventrolateral regions of the AN. However, the number of neurons expressing only TH in the ventrolateral region of the AN (further—ventrolateral AN) was almost three times higher than in the dorsomedial region of the AN (further—dorsomedial AN). In contrast, the vast majority of neurons expressing both dopamine-synthesizing enzymes were concentrated in the dorsomedial AN. Indeed, the number of these neurons in the dorsomedial AN exceeded their number in the ventrolateral AN by 14 times [10].

The functional significance of monoenzymatic neurons remained unclear for a long time until we showed that the neurons expressing individual complementary dopamine-synthesizing enzymes produce this neurotransmitter–neurohormone in cooperation. In this case, L-3,4-dihydroxyphenylalanine (L-DOPA) secreted by monoenzymatic TH-containing neurons is captured by monoenzymatic neurons containing AADC, where dopamine is synthesized. Dopamine can also be synthesized by bienzymatic (non-dopaminergic) neurons lacking the dopamine transporter [9].

When studying AN neurons, it should be given that their functioning and, apparently, development could be controlled by noradrenergic neurons of the brain stem [5,9,11,12,13]. Thus, the aim of this study was to determine whether there is noradrenergic regulation of TH expression in neurons of the ventrolateral and dorsomedial AN in rats during the postnatal period of ontogenesis in young rats (5th postnatal day, P5) and adult rats (60th postnatal day, P60). To meet this aim, the following tasks were set: (i) to evaluate the expression of genes encoding dopamine-synthesizing enzymes—TH and AADC, in the ventrolateral and dorsomedial AN in intact rats (P5, P60); (ii) to determine the change in the TH content in the ventrolateral and dorsomedial AN of rats (P5, P60) after incubating in Krebs–Ringer solution (KRS) for 6 h, which is considered an indicator of TH synthesis; (iii) to evaluate the expression of adrenoreceptor genes in the ventrolateral and dorsomedial AN in intact rats (P5, P60); (iv) to assess whether noradrenaline affects TH synthesis in the ventrolateral and dorsomedial AN of rats (P5, P60) during 6h incubation; and (v) to identify adrenoreceptors in the ventrolateral and dorsomedial AN of rats (P5, P60) involved in the regulation of TH synthesis, over the course of their 6h incubation with or without noradrenaline, as well as with noradrenaline with adrenoreceptor antagonists.

## 2. Results

### 2.1. Gene Expression of Tyrosine Hydroxylase, Aromatic L-Amino Acid Decarboxylase, and Adrenoreceptors in the Ventrolateral and Dorsomedial Regions of the Arcuate Nucleus in Rats on Postnatal Days 5 and 60

**Figure 1:**
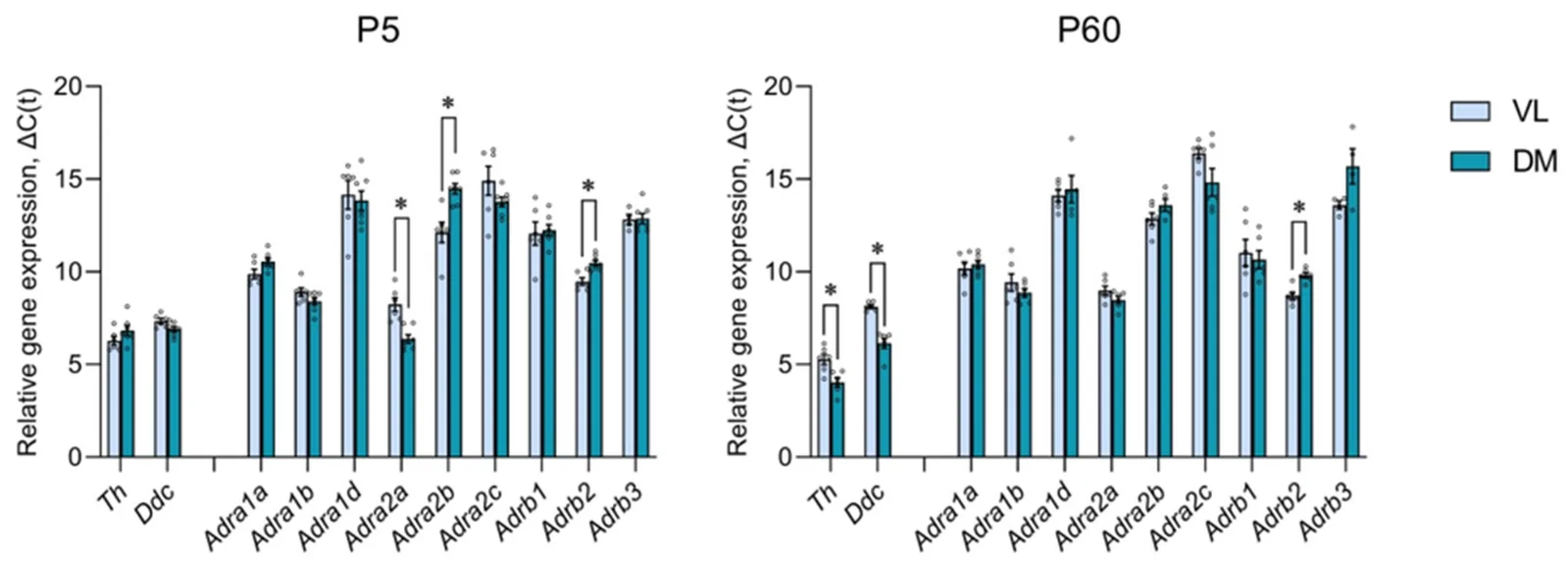
Comparison of the gene expression for tyrosine hydroxylase (*Th*), aromatic L-amino acid decarboxylase (*Ddc*), and adrenoreceptors (*Adra1a*, *Adra1b*, *Adra1d*, *Adra2a*, *Adra2b*, *Adra2c*, *Adrb1*, *Adrb2*, and *Adrb3*) in the ventrolateral (VL) and dorsomedial (DM) regions of the arcuate nucleus in rats on postnatal day 5 (P5) and postnatal day 60 (P60). The normality of the data distribution was assessed using the Shapiro–Wilk test. Statistical significance was determined using an unpaired *t*-test for normally distributed data and the Mann–Whitney U test for non-normally distributed data (* *p* ≤ 0.05). The data are presented as mean ± SEM. Dots indicate individual biological replicates (n = 4–7).

On P5, expression of all the genes under study was detected in the ventrolateral and dorsomedial AN in intact rats, as isolated by laser microdissection (Figure S1). In the ventrolateral AN, gene expression varied as follows, from the most highly expressed gene to the least highly expressed gene: *Th*, *Ddc*, *Adra2a*, *Adra1b*, *Adrb2*, *Adra1a*, *Adrb1*, *Adra2b*, *Adrb3*, *Adra1d*, and *Adra2c*. In the dorsomedial AN, gene expression levels decreased in the following order: *Adra2a*, *Th*, *Ddc*, *Adra1b*, *Adrb2*, *Adra1a*, *Adrb1*, *Adrb3*, *Adra2c*, *Adra1d*, and *Adra2b*. At the same time, on P5, *Adra2a* expression in the dorsomedial AN was 3.65 times higher than in the ventrolateral AN (*p* = 0.0009), whereas compared with the dorsomedial AN, the expression levels of *Adra2b* and *Adrb2* in the ventrolateral AN were 5.13 times (*p* = 0.0022) and 1.99 times (*p* = 0.0022) higher, respectively (Figure 1).

**Figure 2:**
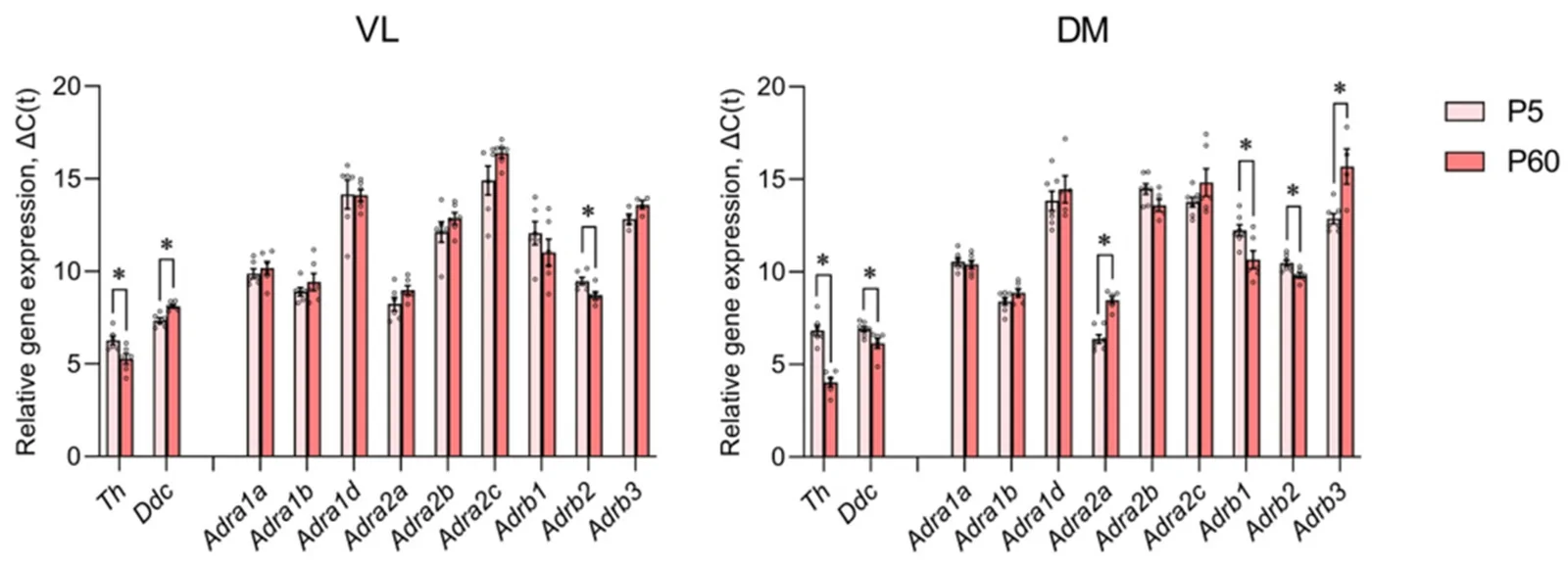
Comparison of the gene expression for tyrosine hydroxylase (*Th*), aromatic L-amino acid decarboxylase (*Ddc*), and adrenoreceptors (*Adra1a*, *Adra1b*, *Adra1d*, *Adra2a*, *Adra2b*, *Adra2c*, *Adrb1*, *Adrb2*, and *Adrb3*) on postnatal days 5 (P5) and 60 (P60) in the ventrolateral (VL) and dorsomedial (DM) regions of the arcuate nucleus in rats. The normality of the data distribution was assessed using the Shapiro–Wilk test. Statistical significance was determined using an unpaired t-test for normally distributed data and the Mann–Whitney U test for non-normally distributed data (* *p* ≤ 0.05). The data are presented as mean ± SEM. Dots indicate individual biological replicates (n = 4–7).

In intact rats on P60, the ventrolateral and dorsomedial AN, as isolated by laser microdissection (Figure S1), expressed the same genes as those observed on P5. In the ventrolateral AN on P60, gene expression varied as follows, from the most highly expressed gene to the least highly expressed gene: *Th*, *Ddc*, *Adrb2*, *Adra2a*, *Adra1b*, *Adra1a*, *Adrb1*, *Adra2b*, *Adrb3*, *Adra1d*, and *Adra2c*. At the same time, in the dorsomedial AN, gene expression levels decreased in the following order: *Th*, *Ddc*, *Adra2a*, *Adra1b*, *Adrb2*, *Adra1a*, *Adrb1*, *Adra2b*, *Adra1d*, *Adra2c*, and *Adrb3* (Figure 1). At the same time, on P60, compared with the ventrolateral AN, the expression of *Th* and *Ddc* in the dorsomedial AN was 2.37 times (*p* = 0.0076) and 3.93 times (*p* = 0.0022) higher, respectively (Figure 2). In contrast, compared with the dorsomedial AN, *Adrb2* expression was 2.15 times higher in the ventrolateral AN at this stage (*p* = 0.001) (Figure 1).

**Figure 3:**
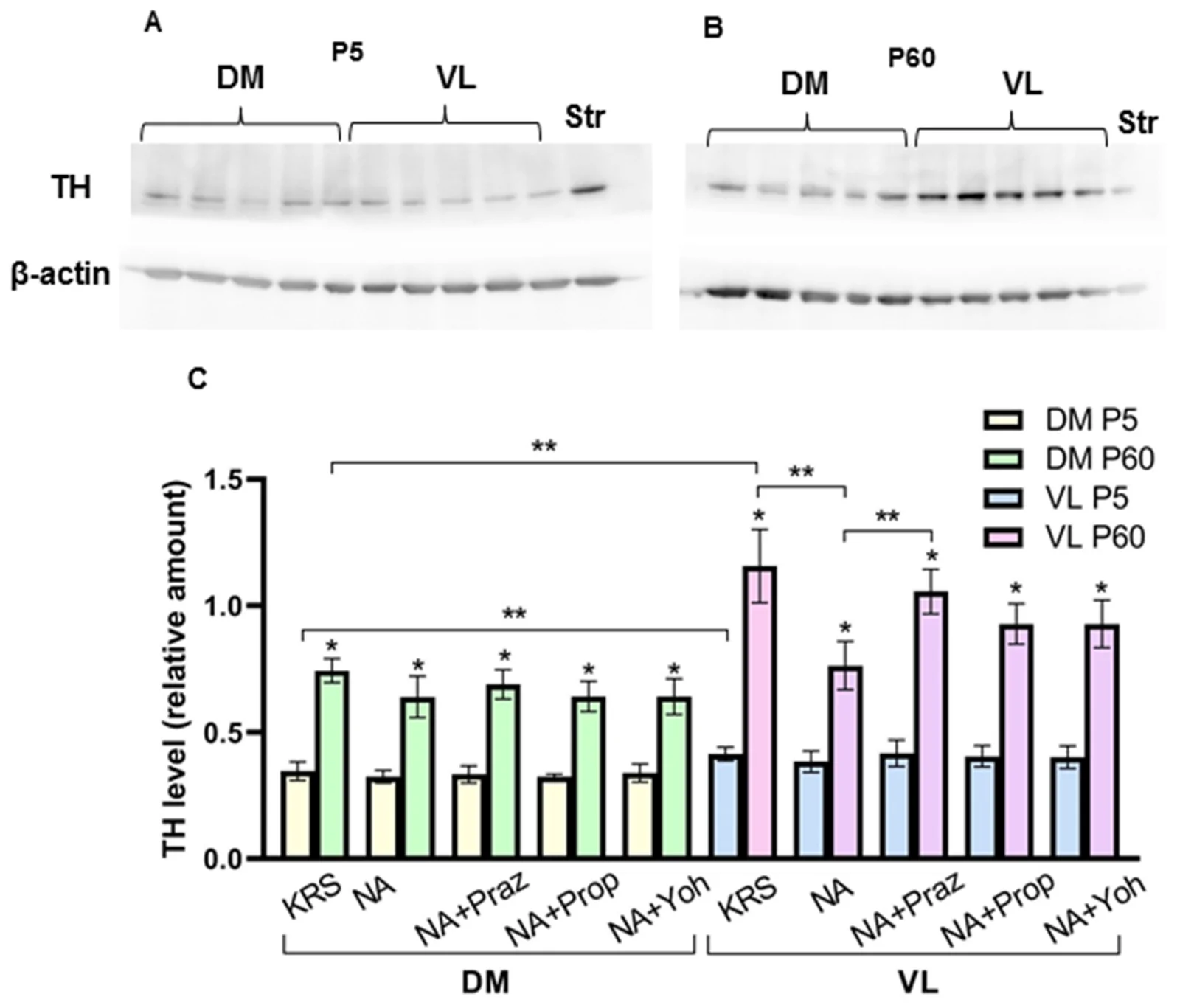
Levels of tyrosine hydroxylase (TH) in the dorsomedial (DM) and ventrolateral (VL) regions of the arcuate nucleus in rats on postnatal days 5 (P5) (**A**,**C**) and 60 (P60) (**B**,**C**), following incubation in Krebs–Ringer Solution (KRS), as well as in KRS supplemented with: (i) 10^−7^ M noradrenaline (NA), (ii) 10^−7^ M NA and 10^−5^ M prazosin (Praz, an α1-adrenoreceptor antagonist), (iii) 10^−7^ M NA and 10^−5^ M propranolol (Prop, a β-adrenoreceptor antagonist), or (iv) 10^−7^ M NA and 5 × 10^−5^ M yohimbine (Yoh, an α2-adrenoreceptor antagonist). The results are presented as mean ± SEM. (n = 6–8) * *p* < 0.05: differences between TH levels on P60 and P5 after equal incubation of the corresponding regions of the arcuate nucleus; ** *p* < 0.05: differences between TH levels in the selected groups.

It was also found that in the ventrolateral AN, *Th* and *Adrb2* expression on P5 was 2 times lower (*p* = 0.02) and 1.71 times lower (*p* = 0.0188), respectively, compared with P60 (Figure 3). In contrast, on P5, *Ddc* expression in the ventrolateral AN was 1.71 times higher (*p* = 0.0011) (Figure 2).

The above results show that the expression of the studied genes at P60 differs significantly from the expression of the same genes at P5. In fact, in the ventrolateral AN at P60 compared to P5: (i) *Th* and *Adrb2* expression was 2-fold (*p* = 0.02) and 1.71-fold (*p* = 0.0188) higher, respectively. In the dorsomedial AN, the expression of *Th*, *Ddc*, *Adrb1*, and *Adrb2* at P60 was 6.97-fold (*p* < 0.0001), 1.69-fold (*p* = 0.0221), 2.99-fold (*p* = 0.0164), and 1.58-fold (*p* = 0.0105) higher, respectively (Figure 2). At the same time, the expression of *Adra2a* and *Adrb3* at P5 in the dorsomedial AN were 4.33 times (*p* < 0.0001) and 7.06 times (*p* = 0.0057) lower, respectively (Figure 2).

### 2.2. Levels of Tyrosine Hydroxylase in the Ventrolateral and Dorsomedial Regions of the Arcuate Nucleus in Rats on Postnatal Days 5 and 60

TH levels were determined by Western blot in different regions of the AN made after incubation in KRS or KRS containing (i) noradrenaline, (ii) noradrenaline and one of the studied adrenoreceptor antagonists, and (iii) the studied adrenoreceptor antagonists and were normalized to TH levels in the rat striatum at P5, which was performed at each electrophoresis.

#### 2.2.1. Postnatal Day 5

In the control group after a 6h incubation of the dorsomedial AN in KRS, the TH level was 1.2 times lower than in the ventrolateral AN (Figure 3).

After a 6h incubation of the dorsomedial and ventrolateral AN in KRS supplemented with noradrenaline (10^−7^ M), TH levels remained unchanged, compared with TH levels following incubation of the dorsomedial and ventrolateral AN in KRS, respectively (Figure 3). However, after incubating the dorsomedial AN in KRS with noradrenaline, the TH level was 1.18 times lower than in the ventrolateral AN (Figure 3).

After a 6h incubation of the dorsomedial and ventrolateral AN in KRS with the addition of noradrenaline (10^−7^ M) and prazosin (10^−5^ M), the TH level did not change, compared with the TH level after incubation of the dorsomedial and ventrolateral AN in KRS with the addition of noradrenaline or incubation in KRS alone, respectively (Figure 3). However, after incubation of the dorsomedial AN in KRS with noradrenaline and prazosin, the TH level was 1.25 times lower than in the ventrolateral AN (Figure 3).

After a 6h incubation of the dorsomedial and ventrolateral AN in KRS supplemented with noradrenaline (10^−7^ M) and propranolol (10^−5^ M), TH levels remained unchanged, compared with TH levels after incubation of the dorsomedial and ventrolateral AN in KRS supplemented with noradrenaline or in KRS alone, respectively (Figure 3). However, after incubation of the dorsomedial AN in KRS with noradrenaline and propranolol, the TH level was 1.25 times lower than in the ventrolateral AN (Figure 3).

After a 6h incubation of the dorsomedial and ventrolateral AN in KRS supplemented with noradrenaline (10^−7^ M) and yohimbine (5 × 10^−5^ M), the TH level did not change compared with the TH level after incubation of the dorsomedial and ventrolateral AN in KRS with the addition of noradrenaline or in KRS alone, respectively (Figure 3). However, after incubation of the dorsomedial AN in KRS with noradrenaline and yohimbine, the TH level was 1.19 times lower than in the ventrolateral AN (Figure 3).

#### 2.2.2. Postnatal Day 60

In the control group, the TH level in the dorsomedial AN in KRS after a 6h incubation was 1.56 times lower than in the ventrolateral AN (Figure 3). Furthermore, on P60, TH levels after a 6h incubation of the dorsomedial and ventrolateral AN in KRS were 2.1 and 2.8 times higher, respectively, than TH levels in the corresponding regions on P5.

After a 6h incubation of the dorsomedial AN in KRS with the addition of noradrenaline (10^−7^ M), the TH level remained unchanged, compared with the TH level following incubation of the dorsomedial AN in KRS (Figure 3). However, after incubating the ventrolateral AN with noradrenaline, the TH level was 34% lower than the TH level in the ventrolateral AN after incubation in KRS. Furthermore, after incubating the dorsomedial AN in KRS with noradrenaline, the TH level was 1.19 times lower than in the ventrolateral AN (Figure 3).

**Figure 4:**
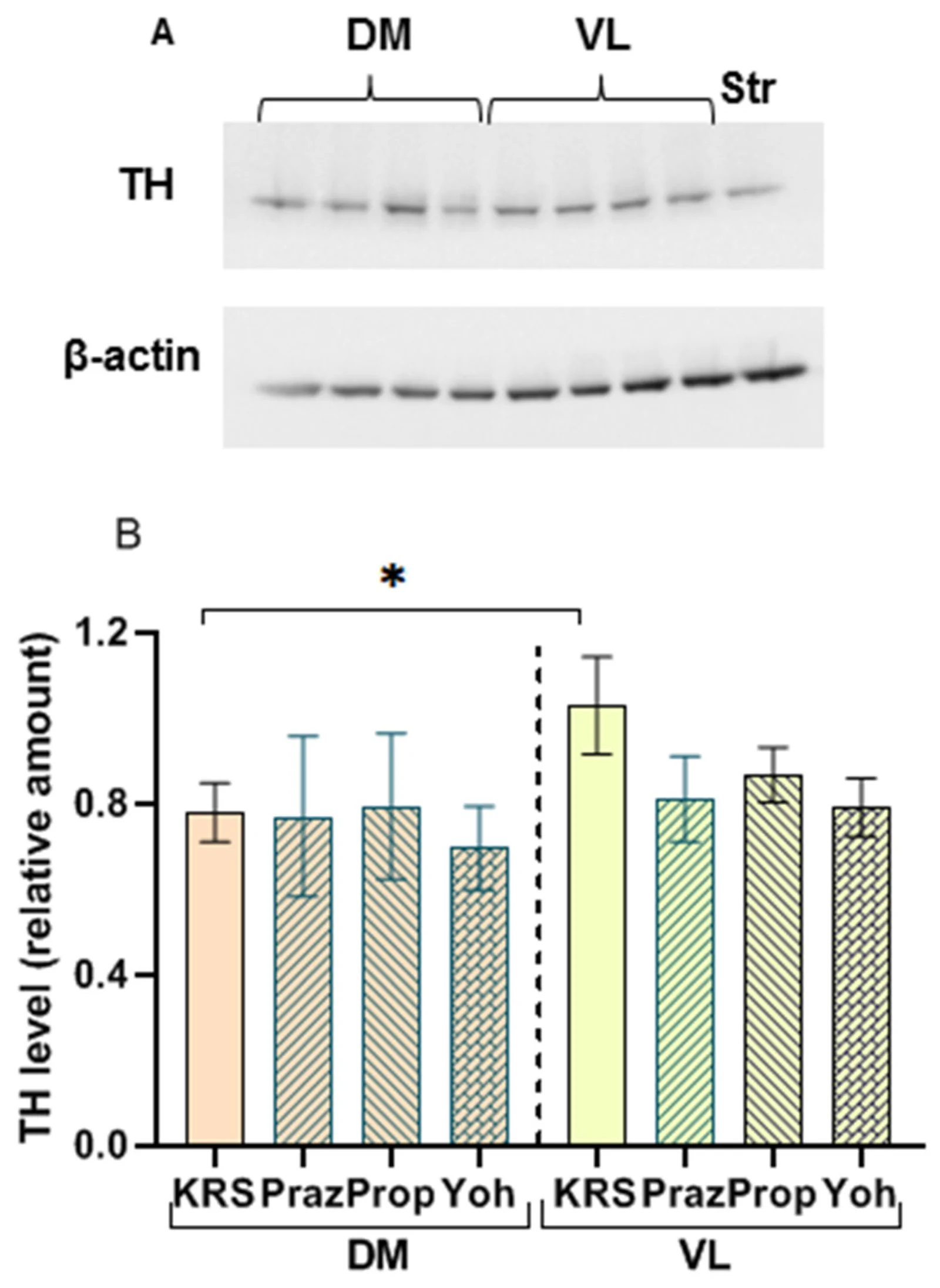
Levels of tyrosine hydroxylase (TH) in the dorsomedial (DM) and ventrolateral (VL) regions of the arcuate nucleus in rats on postnatal day 60 (**A**,**B**), following incubation in Krebs–Ringer Solution (KRS), as well as in KRS supplemented with: (i) 10^−5^ M prazosin (Praz, an α1-adrenoreceptor antagonist, rightward hatching slant), (ii) 10^−5^ M propranolol (Prop, a β-adrenoreceptor antagonist, leftward hatching slant), or (iii) 5 × 10^−5^ M yohimbine (Yoh, an α2-adrenoreceptor antagonist, scaly hatching). The results are presented as mean ± SEM. (n = 5–8) * *p* < 0.05: differences between TH levels in the selected groups.

After a 6h incubation of the dorsomedial AN in KRS supplemented with noradrenaline (10^−7^ M) and prazosin (10^−5^ M), TH levels remained unchanged compared with TH levels following incubation of the dorsomedial AN in KRS supplemented with noradrenaline or in KRS alone (Figure 3). However, after incubating the ventrolateral AN in KRS supplemented with noradrenaline and prazosin, the TH level was 28% higher than the TH level after incubating the dorsomedial AN in KRS supplemented with noradrenaline, and it remained unchanged compared with the TH level after incubation in KRS (Figure 3). Furthermore, after incubating the dorsomedial AN in KRS with noradrenaline and prazosin, the TH level was 1.53 times lower than in the ventrolateral AN following the same procedure (Figure 3). After incubation of the dorsomedial and ventrolateral AN in a KRS with prazosin (10^−5^ M), the TH level remained the same as after incubation of the same regions of the AN in a KRS (Figure 4).

After a 6h incubation of the dorsomedial AN in KRS supplemented with noradrenaline (10^−7^ M) and propranolol (10^−5^ M), TH levels did not change, compared with TH levels after incubation of the dorsomedial AN in KRS supplemented with noradrenaline or in KRS alone (Figure 3). However, after incubation of the ventrolateral AN in KRS supplemented with noradrenaline and propranolol, the TH levels did not change significantly (Figure 3). At the same time, after incubation of the dorsomedial AN in KRS with noradrenaline and propranolol, the TH level was 1.45 times lower than in the ventrolateral AN following the same procedure (Figure 3).

After incubation of the dorsomedial and ventrolateral AN in KRS with propranolol (10^−5^ M), the TH levels remained unchanged compared to TH levels after incubation of the same AN regions in KRS (Figure 4).

After a 6h incubation of the dorsomedial AN in KRS supplemented with noradrenaline (10^−7^ M) and yohimbine (5 × 10^−5^ M), the TH level did not change compared with the TH level after incubation of the dorsomedial AN in KRS supplemented with noradrenaline or in KRS alone (Figure 3). However, after incubation of the ventrolateral AN in KRS supplemented with noradrenaline and yohimbine, TH levels did not change significantly (Figure 3). At the same time, after incubation of the dorsomedial AN in KRS with noradrenaline and yohimbine, the TH level was 1.45 times lower than in the ventrolateral AN following the same procedure (Figure 3). After incubation of the dorsomedial and ventrolateral AN in KRS with yohimbine (5 × 10^−5^ M), TH levels remained the same as after their incubation in KRS (Figure 4).

#### 2.2.3. Differences in TH Levels on P60 and P5

On P60, TH levels after a 6h incubation of the dorsomedial and ventrolateral AN in KRS were 2.14 and 2.79 times higher, respectively, than TH levels in the corresponding regions on P5 (Figure 3). TH levels after incubation of the dorsomedial and ventrolateral AN in KRS with noradrenaline on P60 were 1.97 and 1.99 times higher than TH levels in the corresponding regions on P5 (Figure 3). TH levels after incubation of the dorsomedial and ventrolateral AN in KRS with noradrenaline and prazosin on P60 were 2.07 and 2.53 times higher than TH levels in the corresponding regions on P5 (Figure 3). TH levels after incubation of the dorsomedial and ventrolateral AN in KRS with noradrenaline and propranolol on P60 were 1.98 and 2.29 times higher than TH levels in the corresponding regions on P5 (Figure 3). TH levels after incubation of the dorsomedial and ventrolateral AN in KRS with noradrenaline and yohimbine on P60 were 1.89 and 2.31 times higher than TH levels in the corresponding regions on P5 (Figure 3).

Using the MTT test, the viability of rat AN tissue after 6 h of incubation was confirmed (Figure S2).

## 3. Discussion

The role of monoaminergic innervation of the hypothalamus in regulating neurosecretory neuron differentiation, functioning and contribution to the neuroendocrine regulation has long attracted the attention of researchers [9]. As early as the 1980s, it was shown that deafferentation of the hypothalamus in rats or their salt loading lead to an increase in TH expression in the magnocellular neurons of the supraoptic nucleus [14]. Later, we demonstrated that TH expression in these neurons is inhibited by noradrenaline, which enters the hypothalamus via the axons of noradrenergic neurons in the brainstem [15]. Of particular interest is the fact that inhibitory control of TH expression by noradrenaline in osmotically stimulated vasopressinergic neurons of the supraoptic nucleus is established in rats as early as on P3 [15].

As in our previous study of the development of noradrenergic control of TH expression in supraoptic nucleus neurons [15], in this study we examined noradrenergic control of TH expression in AN neurons in young rats (at P5) during the establishment of noradrenergic innervation and in adult rats (at P60) after the establishment of this innervation. Our study was based on the hypothesis that TH expression in AN neurons, as in supraoptic nucleus neurons, is inhibited by noradrenaline.

It should be noted that the AN contains three populations of neurons expressing TH. Some neurons fully express the dopaminergic phenotype, both dopamine-synthesizing enzymes and the dopamine transporter. Others partially express the dopaminergic phenotype, only TH or only the two dopamine-synthesizing enzymes [9]. Despite certain phenotypic differences, all of these neurons are involved in dopamine synthesis [9].

In this study, changes in TH content in AN after 6h incubation with noradrenaline, or with noradrenaline and adrenoreceptor antagonists, were considered as an indicator of the noradrenergic (noradrenaline) effect on TH synthesis. This indicator is conditional, as the measured TH content in AN neurons included both TH synthesized and degraded over 6 h of incubation and TH contained in neurons prior to incubation. However, we believe that TH synthesis is the most variable, if not the only variable, factor. Although TH is also an attribute of noradrenergic fibers, its content should either remain unchanged, as TH is synthesized outside AN, or decrease due to its enzymatic degradation. In other words, we believe that the contribution of TH contained in noradrenergic fibers can be ignored when assessing the TH synthesis in incubated AN.

To address the main objective of this study, namely, to determine the effect of noradrenaline on TH synthesis in the AN of rats on P5 and P60, sections of the ventrolateral and dorsomedial AN were incubated for 6 h in a KRS (control) or in a KRS with noradrenaline at a concentration of (10^−7^ M).

Using male rats as the study subject, a noradrenaline concentration of 10^−7^ M in the incubation medium, and a 6h slice incubation time, we were guided by a number of considerations. First, given that the AN is the neuroendocrine center regulating reproduction, using female rats would be complicated by the need to determine the phase of the ovulatory cycle and use female rats in the same phase. Then, noradrenaline was used at a concentration of 10^−7^ M, as we believe this concentration is physiologically active. Indeed, it has been shown that classical neurotransmitters, including noradrenaline, exert selective fine neuronal responses in the brain in vivo in physiologically active concentrations ranging from 10^−11^ M to 10^−8^ M. At such low concentrations, neurotransmitters activate highly specific synaptic receptors. To obtain the same selective neuronal response in vitro (slices, cell cultures), neurotransmitters are used at concentrations from 10^−8^ M to 10^−7^ M [16]. At concentrations of 10^−5^ and higher, neurotransmitters exert pharmacological effects by activating all receptor pools, as well as other receptor subtypes with low affinity [16,17,18]. It should be noted that at pharmacological concentrations of neurotransmitters, the sensitivity of the receptors greatly decreases [17]. A six-hour incubation period was chosen for two reasons. On the one hand, this period is sufficient to detect newly synthesized, non-releasable proteins, including TH of AN neurons using Western blotting [15,16]. On the other hand, AN tissue incubated for 6 h remains viable, as confirmed by the MTT assay (Figure S2).

According to the data obtained in this study, at P5, TH content is the same in the ventrolateral and dorsomedial AN, and this level does not change during section incubation with noradrenaline. Theoretically, the lack of effect of noradrenaline on TH synthesis could be the result of opposing actions of noradrenaline through different receptor families. Therefore, we assessed TH synthesis when noradrenaline was applied in combination with its receptor antagonists: prazosin (an α1-adrenoreceptor antagonist), propranolol (a β-adrenoreceptor antagonist), and yohimbine (an α2-adrenoreceptor antagonist). However, even in this case, no change in TH content in the AN was observed. Taken together, these data indicate the absence of noradrenergic control over TH expression in AN neurons in young rats (P5), when noradrenergic innervation of the hypothalamus has not yet fully developed.

According to our data, TH levels in both studied regions of the AN increased severalfold on P60 compared to P5. It is noteworthy that the response to noradrenaline administration in terms of changes in TH levels in neurons of the ventrolateral and dorsomedial AN differed significantly. The TH content in the dorsomedial AN did not change in response to noradrenaline, nor to noradrenaline combined with antagonists of its receptors. This suggests that TH-containing neurons in the dorsomedial AN on P60, like TH neurons throughout the AN on P5, are not sensitive to noradrenaline. This hypothesis is indirectly supported by the relatively low level of expression of adrenoreceptor genes found in this study. In contrast to neurons located in the dorsomedial AN, TH levels in neurons of the ventrolateral AN were significantly reduced by noradrenaline. It means that noradrenaline inhibits TH synthesis specifically in ventrolateral AN, containing the largest number of monoenzymatic TH neurons [9].

Given that the action of noradrenaline on target neurons is mediated through several families of adrenoreceptors, we sought to determine which receptor subtypes mediate the action of noradrenaline on TH synthesis in neurons of the ventrolateral AN in adult rats (P60). To this end, we incubated the ventrolateral and dorsomedial AN with noradrenaline and with noradrenaline in combination with one of the adrenoreceptor antagonists: prazosin (an α1-adrenoreceptor antagonist); propranolol (a β-adrenoreceptor antagonist); and yohimbine (an α2-adrenoreceptor antagonist). It was found that the addition of only prazosin to the incubation medium containing noradrenaline prevented the noradrenaline-induced increase in TH levels. This fact suggests that noradrenaline, via α1-adrenergic receptors, inhibits TH synthesis in neurons of the ventrolateral AN in adult rats (P60). Since prazosin is an antagonist of all α1-adrenoreceptor subtypes and partially α2b- and α2c-adrenoreceptors, and that α1a- and α1b-adrenoreceptors predominate in the ventrolateral AN, it is assumed that these receptors mediate the inhibitory effect of noradrenaline on the synthesis of TH or the stimulating effect on its degradation.

Thus, the data obtained in this study support our hypothesis about the inhibitory effect of noradrenaline on TH synthesis in AN neurons in rats, and this effect was age- and region-specific.

## 4. Materials and Methods

### 4.1. Animals

**Figure 5:**
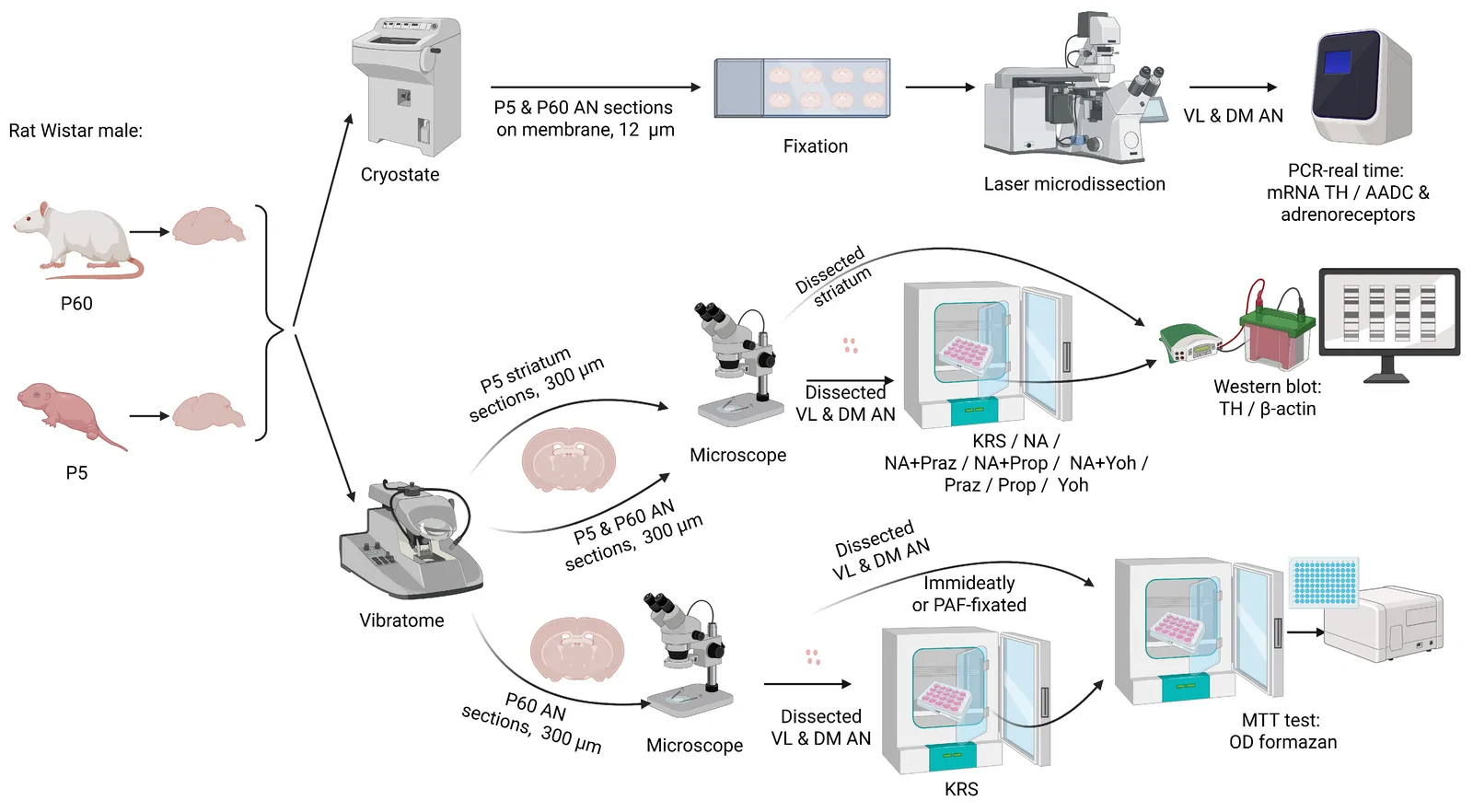
Schematic representation of the methodology used to study the arcuate nucleus (AN) in Wistar rats at postnatal days 5 (P5) and 60 (P60): (1) laser microdissection of dorsomedial (DM) and ventrolateral (VL) areas of AN from 12 μm cryostat sections with subsequent polymerase chain reaction (PCR) of tyrosine hydroxylase (TH), aromatic L-amino acid decarboxylase (AADC) and adrenoreceptors genes; (2) preparation of 300 μm-thick VL and DM AN vibratome sectionsfollowed by Western blotting to assess tyrosine hydroxylase levels (synthesis) after 6 h incubation in Krebs–Ringer solution (KRS) or KRS with norepinephrine (NA) or NA with prazosin (Praz)/propranolol (Prop)/yohimbine (Yoh) or KRS with prazosin (Praz)/propranolol (Prop)/yohimbine (Yoh); (3) preparation of 300 μm-thick vibratome sections of VL and DM AN followed by 3-(4,5-dimethylthiazol-2-yl)-2,5-diphenyl-tetrazolium bromide (MTT) test to assess tissue viability; pre-incubation with paraformaldehyde was used as a negative control. Beige dots, dissected VL&DM AN.

Wistar male rats on postnatal days 5 (P5) and 60 (P60) were used in this study. The day of birth was considered the first postnatal day (Figure 5).

The animals were housed under standard laboratory conditions in the vivarium: at a temperature of 21–23 °C, on a 12h light-dark cycle, and with free access to food and water.

### 4.2. Immunohistochemistry and Microscopy

Rats were anesthetized with chloral hydrate (intraperitoneally, 400 mg/kg) and were perfused through the heart first with a 0.9% NaCl solution in 0.02 M phosphate-buffered saline (PBS) (pH 7.2–7.4) at 37 °C for 15 min, and then with a 4% paraformaldehyde solution in 0.1 M phosphate buffer (pH 7.2–7.4) at 4 °C for 15 min. After perfusion fixation, the rats were decapitated, and the removed brains were fixed by immersion in 4% paraformaldehyde in 0.1 M phosphate buffer (pH 7.2–7.4) for 12 h at 4 °C. The brains were then sequentially washed in PBS, incubated in 20% sucrose in PBS at 4 °C for 24 h, frozen at –40 °C, and stored at –70 °C until immunocytochemistry was performed.

For immunocytochemistry, serial frontal brain sections, 16 µm thick, were prepared at the level of the AN using a cryostat (Leica CM1950, Leica, Wetzlar, Germany). Every fifth section was mounted on slides and sequentially incubated in PBS containing: (i) 3% bovine serum albumin (Sigma, St. Louis, MO, USA) and 0.3% Triton X-100 (Sigma, St. Louis, MO, USA) for 1 h at 20 °C; (ii) sheep polyclonal antibodies to tyrosine hydroxylase (TH) (1:700) (Millipore, Burlington, MA, USA, AB 1542), rabbit polyclonal antibodies to AADC (1:400) (Synaptic Systems GmbH, Goettingen, Germany), 1% bovine serum albumin, and 0.1% Triton X-100 for 20 h at 20 °C; (iii) Alexa-Fluor-555 donkey antibodies against rabbit gamma globulins (1:1000) (Invitrogen, Thermo Fisher Scientific, Waltham, MA, USA, A32794) for 2 h at 20 °C; and (iv) Alexa-Fluor-488 donkey antibodies against sheep gamma globulins (1:1000) (Invitrogen, Thermo Fisher Scientific, Waltham, MA, USA, A11015) for 2 h at 20 °C. After each incubation, except for the first, the sections were washed three times in PBS for a total of 45 min. After the last incubation, the sections were washed in PBS for one hour and mounted in a medium containing DAPI (Abcam, Cambridge, UK).

AN sections immunostained for TH and AADC were examined under a fluorescence microscope (Leica DMi8 M, Leica Camera AG, Wetzlar, Germany) at a 20× objective magnification (The Core Center of the Koltzov Institute of Developmental Biology, Russian Academy of Sciences) (Figure S1).

### 4.3. Laser Microdissection of the Ventrolateralandand Dorsmedial Regions of the Arcuate Nucleus

Rats were decapitated under isoflurane anesthesia (LaboratoriosKarizoo, Barcelona, Spain) using a SomnoSuite anesthesia machine (Kent Scientific, Torrington, CT, USA), and their brains were isolated. A 15 mm × 15 mm × 15 mm block containing the AN was then excised from the brain, frozen in liquid nitrogen, and stored at −70 °C [19]. The cryostat chamber and instruments were pretreated with 6% hydrogen peroxide and 96% ethanol and were irradiated with UV light for 2.5 h. Polyester membranes with 0.9 μm-sized pores (POL-membrane, Frame Slides, Leica, Germany) were pretreated for 5 min with RNAseClean solution (BioinnLabs, Rostov-on-Don, Russia), were washed twice with diethylpyrocarbonate-treated water (Evrogen, Moscow, Russia) for 30 s each, were then washed twice with 96% ethanol for 30 s each, and were dried in a laminar flow hood under UV light for 45 min. The treated membranes were stored in a closed dry chamber at 20 °C until section mounting.

The12µm-thick serial frontal brain sections were then cut in a cryostat (Leica CM1950, Leica, Wetzlar, Germany) at the level of the AN and were mounted onto a membrane. Immediately after preparation, the cryostat brain sections were fixed for 3 min in Carnoy’s fixative solution (60% ethanol + 30% chloroform + 10% glacial acetic acid (all: Sigma, St. Louis, MO, USA)) at −20 °C. From the fixed sections, using laser microdissection (Leica LMD7000, Wetzlar, Germany), the ventrolateral and dorsomedial AN were excised separately (Figure S1). Laser microdissection was performed under microscopic control at a 5x objective magnification, power 40, aperture 40, speed 55, and pulse frequency 910. Finally, the excised tissue fragments were collected in the lids of 0.6 mL Eppendorf tubes containing 50 µL of TRI Reagent (Sigma-Aldrich, St. Louis, MO, USA). The prepared samples were frozen in liquid nitrogen and stored at −70 °C until real-time polymerase chain reaction (PCR).

### 4.4. RNA Extraction, and PCR

For RNA extraction and subsequent PCR, the volume of TRI-reagent containing the excised AN fragments was adjusted to 1 mL, and 100 µL of 1-bromo-3-chloropropane (Sigma-Aldrich, St. Louis, MO, USA) was added. This solution was vortexed and incubated for 15 min at 20 °C, and the phases were then separated by centrifuging the solution for 15 min at 21,000× *g* and 20 °C. The aqueous phase containing RNA was transferred to a new tube, to which 500 µL of isopropyl alcohol (Sigma-Aldrich, St. Louis, MO, USA) and 0.5 µL of glycogen (Thermo Fisher Scientific, Waltham, MA, USA) were added for RNA precipitation. The solution was mixed and incubated for 10 min at 20 °C, after which the RNA was precipitated for 20 min at 21,000× *g* and 4 °C. The supernatant was removed, and the precipitate was washed three times with 1 mL of 80% ethanol and centrifuged for 10 min at 21,000× *g* and 4 °C. After the final centrifugation, the ethanol was removed, and the precipitated RNA was air-dried for 15 min and diluted in 9 µL of RNase- and DNase-free water. The concentration and purity of the RNA in the prepared samples were measured using a NanoDrop 8000 spectrophotometer (Thermo Fisher Scientific, Waltham, MA, USA), and only samples with A260/280 ratios of 1.8–2.1 and A260/230 ratios of 1.8–2.2 were processed further.

RNA concentration and purity were assessed using a NanoDrop 8000 spectrophotometer (Thermo Fisher Scientific, Waltham, MA, USA), and only samples with A260/280 ratios of 1.8–2.1 and A260/230 ratios of 1.8–2.2 were processed further. Residual genomic DNA was removed using DNase I RNase-free (Thermo Fisher Scientific, Waltham, MA, USA) according to the manufacturer’s recommendations. Complementary DNA was synthesized from 150 ng of total RNA using a Maxima H Minus First Strand complementary DNA synthesis kit (Thermo Fisher Scientific, Waltham, MA, USA), according to the manufacturer’s recommendations. The concentration of complementary DNA was measured using a NanoDrop 8000 spectrophotometer.

Quantitative polymerase chain reaction was performed in a QuantStudio 12k Flex thermal cycler (Applied Biosystems, Thermo Fisher Scientific, Waltham, MA, USA), using a qPCRmix-HS SYBR+LowROX reaction mixture (Evrogen, Moscow, Russia) and oligonucleotide primers (Evrogen, Moscow, Russia) (Table 1). Then, 500 ng of complementary DNA was taken into the reaction. The expressions of the *Th*, *Ddc*, and adrenoreceptors genes werecalculated using the ΔC(t) method. *Rpl13a* was used as a reference gene (housekeeping gene).

∆Ct = (Ct(*targetgene*) − Ct(*Rpl13a*),

where C(t) is the cycle threshold value corresponding to the point at which the amplification curve intersects the fixed threshold line.

### 4.5. Incubation of the Ventrolateral and Dorsomedial Arcuate Nucleus with Noradrenaline and Adrenoreceptor Antagonists

Rats were decapitated under isoflurane anesthesia, and their brains were isolated. Serial frontal brain sections, 300 µm thick and containing AN, were then prepared on a vibratome (Leica VT 1200S, Leica, Wetzlar, Germany) in KRS containing the following (mM): NaCl 120, KCl 4.8, CaCl_2_ 2.0, MgSO_4_ 1.2, NaHCO_3_ 25, D-glucose 10.1, HEPES 20, and ascorbic acid 0.1 (pH = 7.4) (all: Sigma, St. Louis, MO, USA), saturated with carbogen (O_2_: 95%, CO_2_: 5%), at 4 °C. In P5 rats, the ventrolateral and dorsomedial AN were dissected from three vibratome sections under a binocular microscope using the atlas by Paxinos and Ashwell [20]. In P60 rats, the ventrolateral and dorsomedial AN were dissected from four vibratome sections under a binocular loupe using the atlas by Paxinos and Watson [21].

The sections of the ventrolateral and dorsomedial AN were sequentially statically incubated: (1) in KRS for 30 min at 37 °C for stabilization; (2) for 6 h at 37 °C in KRS, or in KRS with the addition of the following (replacing the solution every 120 min): (i) 10^−7^ M noradrenaline, (ii) 10^−7^ M noradrenaline and 10^−5^ M prazosin (an α1-adrenoreceptor antagonist), (iii) 10^−7^ M noradrenaline and 5 × 10^−5^ M yohimbine (an α2-adrenoreceptor antagonist), and (iv) 10^−7^ M noradrenaline and 10^−5^ M propranolol (β-adrenoreceptor antagonist), (v) 10^−5^ M prazosin (an α1-adrenoreceptor antagonist), (v) 5 × 10^−5^ M yohimbine (an α2-adrenoreceptor antagonist), and (vi) 10^−5^ M propranolol (β-adrenoreceptor antagonist) (all reagents: Sigma, St. Louis, MO, USA). After incubation, the tissue was frozen in liquid nitrogen and stored at −70 °C until the determination of TH by Western blot.

### 4.6. Western Blotting of Tyrosine Hydroxylase

To each ventrolateral and dorsomedial AN tissue sample, 30 μL of RIPA extraction buffer was added (50 mM Tris-HCl, pH 7.4, 150 mM NaCl, 1% NP-40, 0.5% sodium deoxycholate, and 0.1% sodium dodecyl sulfate (all reagents: Sigma, St. Louis, MO, USA)), and the samples were then homogenized using an ultrasonic homogenizer at 4 °C. The striatum sample was prepared in the same way in 200 μL of RIPA from frontal brain sections of one rat on P5. The resulting homogenates were centrifuged for 20 min at 20,000× *g* at 4 °C. The protein content in the supernatant was determined using the “BCA Protein Assay Kit” according to the manufacturer’s instructions (Thermo Scientific, Waltham, MA, USA). Then, proteins were denatured for 5 min at 95 °C in 5% β-mercaptoethanol. Before sample loading, Laemmli sample buffer containing 2% sodium dodecyl sulfate, 10% glycerol, 5% β-mercaptoethanol, 62.5 mM Tris (pH 6.8), and 0.004% bromophenol blue was added to each sample in a 1:4 volume ratio. A total of 15 mg of protein from ventrolateral and dorsomedial AN or striatum samples were loaded per lane. Electrophoresis was performed on a 12% polyacrylamide gel. Thereafter, proteins were transferred to a nitrocellulose membrane (Hybond-enhanced chemiluminescence, Amersham Biosciences, Slough, UK) overnight at 30 mA. Protein loading and uniform transfer to the membrane were confirmed by Ponceau-S staining [22,23]. Each membrane was divided into two parts transversely at the level of proteins with a molecular weight of 50–55 kDa. Ponceau-S was then removed by incubating the membranes in Tris-buffered saline (100 mM Tris-HCl, 150 mM NaCl and 0.05% Tween 20) with shaking for 10 min. Nonspecific reaction on the membrane was blocked by incubating it in Tris-buffered saline with 5% bovine serum albumin for 1 h at 20 °C with shaking (Sigma-Aldrich, Saint Louis, MO, USA). To determine TH, the upper parts of the membranes were incubated with primary mouse monoclonal antibodies to TH (1:1000, T1299, Sigma-Aldrich, Saint Louis, MO, USA) for 20 h at 4 °C. The lower parts of the membranes were incubated with β-actin mouse monoclonal antibodies (1:5000; A1978, Sigma-Aldrich, Saint Louis, MO, USA). After washing in Tris-buffered saline to remove primary antibodies, the membranes were incubated with secondary antimouse-Ig antibodies conjugated to horseradish peroxidase for 2 h at 20 °C. Clarity Max Western ECL Substrate (Biorad, Hercules, CA, USA) was used to visualize TH and β-actin according to the manufacturer’s recommendations. The chemiluminescence of the bands was recorded with a ChemiDoc Touch device (Biorad, Hercules, CA, USA) for 1 min. The chemiluminescence intensity was then measured densitometrically using ImageLab software v.3.0 (Biorad, Hercules, CA, USA). TH signals were normalized to β-actin. To ensure uniform scaling of the data across all blots, additional normalization was performed using a striatum of a P5 rat, which was applied to all electrophoresis.

### 4.7. MTT Test

To verify the viability of the AN tissue after 6 h of incubation, the MTT test [24,25] was performed. For this purpose, the ventrolateral and dorsomedial AN were dissected from vibratome sections of 3 rats at P60, as described previously (Section 4.5). Two samples of each AN region were incubated statically, first in KRS for 30 min at 37 °C for stabilization and then in KRS (replacing the solution every 120 min) for 6 h at 37 °C. Two samples of each AN region were collected immediately before the MTT assay (incubation with formazan) and before the MTT assay but after preliminary fixation in 4% paraformaldehyde in 0.1 M phosphate buffer (pH 7.2–7.4) at 4 °C for 30 min, and then washed three times in 0.02 M phosphate-buffered saline for 10 min. All samples were weighed and placed in separate wells of a 96-well plate in 90 μL of Dulbecco’s modified Eagle’s medium (Servicebio, Wuhan, Hubei Province, China) with 25 mM HEPES (Paneco Ltd., Moscow, Russia). Then, 10 μL of a 5 mg/mL MTT were added to each well (3-(4,5-Dimethyl-2-thiazolyl)-2,5-diphenyl-2H-tetrazolium bromide, Paneco Ltd., Moscow, Russia) stock in Hanks’ Balanced Salt Solution (Paneco Ltd., Moscow, Russia). After 2 h of incubation at 37 °C, the MTT solution was removed from the wells. Then, 100 µL of dimethyl sulfoxide was added to each well to dissolve the crystals of formazan, a product of MTT oxidation resulting from the functioning of living mitochondria, and this solution was incubated for 15 min at 37 °C on a shaker. The resulting solution was transferred to new wells of a 96-well plate to assess the optical density. Wells with dimethyl sulfoxide without tissue samples were used as a blank standard. Optical density (OD) of the obtained formazan solution was measured at 540 nm and 630 nm as a reference wavelength using a microplate reader (iMark, Hercules, CA, USA, BioRad). The results were normalized to the sample weight. The MTT assay showed good tissue preservation after incubation (Figure S2).

### 4.8. Statistical Analysis

Statistical analysis was performed using Prism 9.5.1 (GraphPad Software, San Diego, CA, USA). The type of distribution was assessed using the Shapiro–Wilk test. For pairwise comparisons, the paired t-test was used. For parametric multiple comparisons, a one-way ANOVA and posthoc Tukey test were used. For nonparametric pairwise multiple comparisons, Dunn’s test was used. The results are presented as mean ± SEM or as the mean with interquartile range. Differences were considered significant at *p* ≤ 0.05.

## 5. Conclusions

It was shown that:

(1) Neurons in the ventrolateral and dorsomedial AN synthesize many times more TH in adult animals than young ones.
(2) Neurons in the ventrolateral and dorsomedial AN of young animals, as well as neurons in the dorsomedial AN of adult animals, are not sensitive to noradrenaline.
(3) Noradrenaline inhibits TH synthesis in neurons of the ventrolateral AN in adult animals.
(4) The effect of noradrenaline to TH-expressing neurons in the ventrolateral AN is mediated at least in part through α1-adrenoreceptors: apparently α1a- and α1b-adrenoreceptors.

## Figures and Tables

**Table 1:** Oligonucleotide primers used for qPCR.

Gene	Forward Primer	Reverse Primer
*Th*	AGAGGGATGGGAATGCTGTT	GGCCGGGTCTCTAAGTGGT
*Ddc*	CCCCAGGAGCCAGAAACA	AAGCCAATGCAGCCGATAG
*Adra1a*	CATCGGACCCCTGTTCG	GGATGCGGAGCGTCACT
*Adra1b*	CGGACGCCCACCAACTA	AGGCCGTACAGCACAGGAC
*Adra1d*	CCAGCCTGTCCCATAAGATTC	TAGTCGCCCGGCTCATAAG
*Adra2a*	GTTCCCGTTCTTTTTCACCTAC	ACGATGCGCTTTCTGTCC
*Adra2b*	GTGAGCCGAGCATTGGAGTA	TGGTCGCCCTTGTAGATGAG
*Adra2c*	TTCCCGCCTCTCGTCTCT	CTTGCCCAACCCATTCTCT
*Adrb1*	CGTCCGTCGTCTCCTTCTAC	GCAGCTGTCGATCTTCTTCAC
*Adrb2*	AAAGGCAGCTGCAGAAGATAGA	CAGCCAGCAGAGGGTGAAG
*Adrb3*	GCTGAGGCGCAAGAGTGT	AAGGAGGGGAAGGTAGAAGG
*Rpl13a*	GCCGAAAGGTGGTGGTT	GGGGCTCGGAAGTGGTA

## Data Availability

The data presented in this study are available upon request from the corresponding author. The data are not publicly available due to legal issues.

## Supplementary Materials

The following supporting information can be downloaded at: https://www.mdpi.com/article/10.3390/ijms27167308/s1.

## Abbreviations

The following abbreviations are used in this manuscript:

AADC	aromatic L-amino acid decarboxylase
AN	arcuate nucleus
KRS	Krebs–Ringer solution
P	postnatal day
TH	tyrosine hydroxylase
