# Supplementary material for: Noradrenaline Regulation of Tyrosine Hydroxylase Expression in Arcuate Nucleus Neurons in Young and Adult Rats
Source: Int J Mol Sci. 2026 Aug 16;27(16):7308. doi: 10.3390/ijms27167308 (PMC13513824; doi:10.3390/ijms27167308)
Supplement: Supplementary file 1 [file ijms-27-07308-s001.zip › ijms-4470138-supplementary.pdf]

Technical division of the arcuate nucleus.

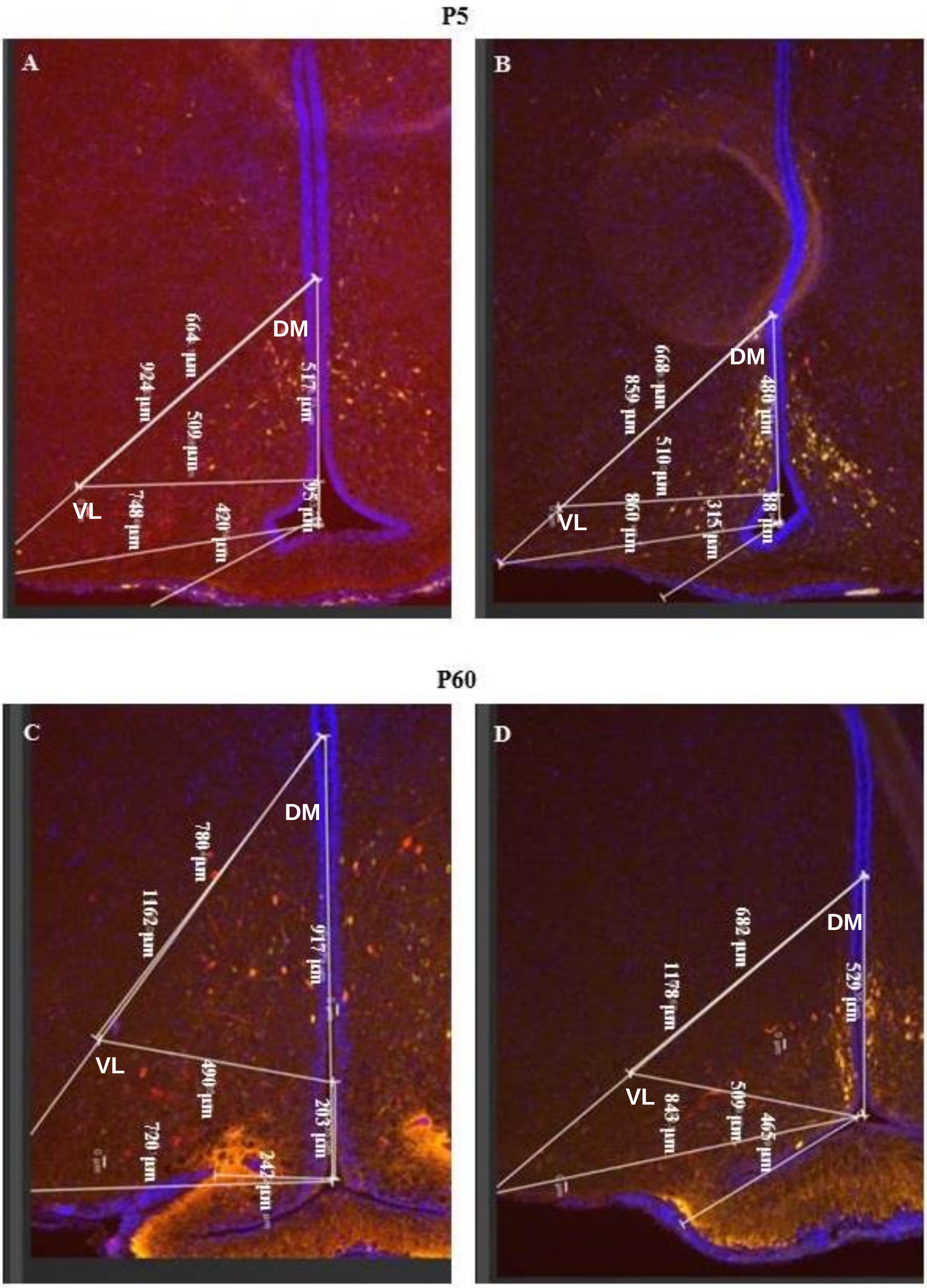

Figure S1. Tyrosine hydroxylase- and aromatic L-amino acid decarboxylase-immunopositive-neurons in the arcuate nucleus of Wistar male rats on postnatal day 5 (A, B) and 60 (C, D). Technical division into ventrolateral (VL) and dorsomedial (DM) regions of the arcuate nucleus.

Determination of tissue viability after incubation in Krebs-Ringer solution using the MTT test.

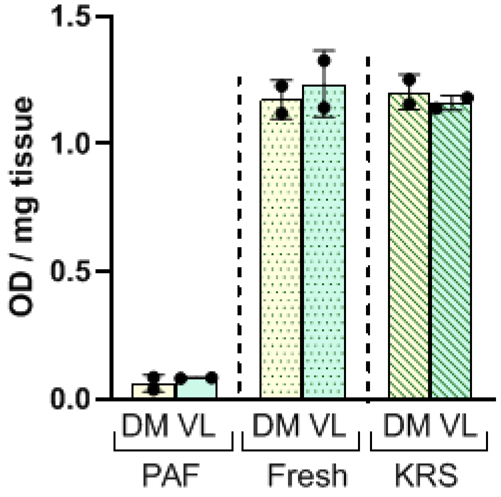

Figure S2. Optical density of formazan solution (an oxidation product of MTT) after incubation of the dorsomedial (DM) and ventrolateral (VL) regions of the arcuate nucleus of rats on postnatal day 60: (i) 6 h in Krebs-Ringer solution (KRS), then 2 h in MTT solution, (ii) 2 h in MTT solution (Fresh, positive control), (iii) 30 min in 4% paraformaldehyde (PAF), then 2 h in MTT solution (negative control). The data are presented as mean ± SD. Dots indicate individual biological replicates.
